# Perinatal outcomes of Aboriginal women with mental health disorders

**DOI:** 10.1177/00048674231160986

**Published:** 2023-03-16

**Authors:** Akilew A Adane, Carrington CJ Shepherd, Roz Walker, Helen D Bailey, Megan Galbally, Rhonda Marriott

**Affiliations:** 1 Ngangk Yira Institute for Change, Murdoch University, Murdoch, WA, Australia; 2 Telethon Kids Institute, The University of Western Australia, Nedlands, WA, Australia; 3 Curtin Medical School, Faculty of Health Sciences, Curtin University, Perth, WA, Australia; 4 School of Indigenous Studies, The University of Western Australia, Crawley, WA, Australia; 5 School of Clinical Health Sciences, Faculty of Medicine, Nursing and Health Sciences, Monash University, Clayton, VIC, Australia

**Keywords:** Aboriginal, mental disorder, preterm birth, low birthweight, perinatal death, linked data

## Abstract

**Objective:**

Maternal mental disorders have been associated with adverse perinatal outcomes such as low birthweight and preterm birth, although these links have been examined rarely among Australian Aboriginal populations. We aimed to evaluate the association between maternal mental disorders and adverse perinatal outcomes among Aboriginal births.

**Methods:**

We used whole population-based linked data to conduct a retrospective cohort study (*N* = 38,592) using all Western Australia singleton Aboriginal births (1990–2015). Maternal mental disorders were identified based on the International Classification of Diseases diagnoses and grouped into six broad diagnostic categories. The perinatal outcomes evaluated were preterm birth, small for gestational age, perinatal death, major congenital anomalies, foetal distress, low birthweight and 5-minute Apgar score. We employed log-binomial/-Poisson models to calculate risk ratios and 95% confidence intervals.

**Results:**

After adjustment for sociodemographic factors and pre-existing medical conditions, having a maternal mental disorder in the five years before the birth was associated with adverse perinatal outcomes, with risk ratios (95% confidence intervals) ranging from 1.26 [1.17, 1.36] for foetal distress to 2.00 [1.87, 2.15] for low birthweight. We found similar associations for each maternal mental illness category and neonatal outcomes, with slightly stronger associations when maternal mental illnesses were reported within 1 year rather than 5 years before birth and for substance use disorder.

**Conclusions:**

This large population-based study demonstrated an increased risk of several adverse birth outcomes among Aboriginal women with mental disorders. Holistic perinatal care, treatment and support for women with mental disorders may reduce the burden of adverse birth outcomes.

## Introduction

Mental health problems, both symptoms and disorders, are more prevalent in Australian Aboriginal and Torres Strait Islander (hereafter Aboriginal) than non-Aboriginal populations, with around a four-fold elevated risk of any mental health problems and up to 10 times greater risk for substance use disorders (Nasir et al., 2018; O’Leary et al., 2013; Ogilvie et al., 2021). Moreover, limited evidence suggests a high prevalence or risk of mental health disorders in Aboriginal mothers (Bowen et al., 2013; Lima et al., 2019; Owais et al., 2020). The higher burden of mental health problems in the Aboriginal population is a legacy of the post-colonial history of Australia and the associated ongoing effects of intergenerational trauma, interpersonal and structural racism, socio-economic deprivation and systematic discrimination, and the greater burden of physical health conditions (Ban et al., 2012; Dudgeon and Walker, 2022; Kelaher et al., 2014; Menzies, 2019; Shepherd et al., 2017). Maternal mental health problems have been associated with several adverse perinatal outcomes such as preterm birth and low birthweight (Montagnoli et al., 2020), which are more common among the Aboriginal than non-Aboriginal population (AIHW, 2021; Ford et al., 2018; Smylie et al., 2010).

Despite the higher burden of mental health problems among Aboriginal women and their potentially serious impacts (Owais et al., 2020; Pierce et al., 2020), scarce empirical evidence exists about the link between maternal mental health problems and perinatal outcomes. A study in Western Australia (WA) examined a range of perinatal and child outcomes among births to Aboriginal and non-Aboriginal women with alcohol use disorder (O’Leary et al., 2020). It highlighted that maternal alcohol use disorder was significantly associated with a greater risk of small for gestational age (SGA) and preterm birth (for both populations) and congenital anomalies and foetal alcohol syndrome (for Aboriginal women only). Although the study provided critical evidence, it was limited to alcohol use disorder and did not evaluate perinatal mortality. Meta-analyses and systematic reviews in the mainstream population also show that women with mental health problems are 1.5–1.9 times more likely to have a preterm birth, low birthweight and perinatal death (Adane et al., 2021; Simonovich et al., 2021; Staneva et al., 2015), although the majority of studies have only focused on maternal anxiety and depression. The underlying mechanisms linking maternal mental health problems and perinatal outcomes are not fully understood but are likely to be complex, vary by disorder and involve altered intrauterine (e.g. elevated cortisol levels) and behavioural (e.g. a reduction in the mother’s ability or attention to gauge what should or should not be done during pregnancy) pathways (Aktar et al., 2019; Pearson et al., 2012).

A better understanding of the relationship between Aboriginal maternal mental health disorders and perinatal outcomes can guide public health policies, prevention strategies and clinical practices to target health equity approaches. Accordingly, this study sought to comprehensively examine the relationship between a range of mental health disorders among Aboriginal women before or during pregnancy and the perinatal outcomes of their offspring.

## Methods

### Study design and data source

A retrospective population-based cohort study was conducted using all WA singleton Aboriginal births from 1990 to 2015. Seven datasets obtained from routinely linked WA databases were used, namely the Hospital Morbidity Data Collection (HMDC), Mental Health Information System (MHIS), Midwives Notification System (MNS), WA Register of Developmental Anomalies, the Derived Aboriginal Status Flag and the WA Registry of Births, Deaths and Marriages. The MNS records the circumstance of all WA births (since 1980) of 20 weeks’ or more gestation or with a birthweight of ⩾400 g; this was used to establish the study cohort. The HMDC includes information on admissions to public and private acute hospitals, public and private psychiatric hospitals, and private day surgeries. The MHIS collects data from community residential facilities, acute general hospitals, psychiatric inpatient units and clinics and psychiatric day centres. We used the WA Registries of Births and Deaths data to supplement the information obtained from the MNS and identity deaths in the first month of life. The WA Register of Developmental Anomalies was used to identify births with major congenital anomalies. The Derived Aboriginal Status Flag is a variable created by the WA Department of Health Data Linkage Branch by applying a validated algorithm (Christensen et al., 2014) across a wide range of administrative datasets to produce a single indicator of Aboriginal status. This algorithm was developed as part of the ‘Getting Our Story Right’ project to improve information about Aboriginal status. The WA Department of Health Research Data Services team linked these datasets together using probabilistic matching techniques (Holman et al., 1999) and securely transferred the data (with identifying fields removed) to the research team.

### Maternal mental health disorders

Data on maternal mental health disorders were obtained from the HMDC and the MHIS. Maternal mental health disorders were identified using ICD-10-AM diagnosis codes (International Statistical Classification of Diseases and Related Health Problems Tenth Edition, Australian Modification) and mapping tables for ICD-9-AM. These data were linked to the MNS. Women were considered to have a mental health disorder if they had a primary or additional mental health ICD diagnosis in the HMDC or MHIS datasets in 5 years preceding the index birth (the birth event included in the analysis). They were classified into broad diagnostic categories (severe mental disorder, common mental disorder, personality disorder, substance use disorder and all other adulthood- and childhood-onset mental disorders). Severe mental disorder included schizophrenia or related disorders, schizoaffective disorders, psychotic affective disorders (including bipolar disorders) and psychotic disorders related to substance use. Common mental disorder included non-psychotic affective disorders and neurotic and stress-related (anxiety) disorders. A woman could have more than one singleton birth during the study period, and these births could vary in the maternal mental disorder exposure status. Supplementary Table S1 describes the mental disorder diagnostic categories and related ICD codes. Women could contribute to more than one diagnostic category if they received more than one psychiatric diagnosis during the study period. Additionally, women were grouped into those who had any mental health diagnosis (at least one of the above-listed disorders) or not. Based on the timing (in relation to childbirth), maternal mental health disorders were considered to occur in 1 or 5 years preceding the index birth.

### Perinatal outcomes

Preterm birth (<37 weeks’ gestation), SGA and perinatal death (including stillbirth and neonatal death) were the primary outcomes of interest. Neonatal death was defined based on the ‘date of birth’ and ‘date of death’ variables obtained from the WA Registers of Births and Deaths, respectively. As we had month and year of birth information only, we created two versions of the neonatal death variable: (1) including deaths that occurred within the same month and year of birth and those reported in the month immediately following the birth month; and (2) including deaths that reported only within the same month and year of birth. The latter was only used in sensitivity analyses. Australian national singleton birthweight centiles (Dobbins et al., 2012), which account for sex and gestation, were used to define SGA (<10th percentile). Preterm birth and SGA were combined to create four mutually exclusive categories: term appropriate for gestational age (term-AGA), term SGA (term-SGA), preterm AGA (preterm-AGA) and preterm SGA (preterm-SGA). Other outcome variables were major congenital anomalies, low birthweight, low Apgar score at 5 minutes (low Apgar-5) and foetal distress (listed as a complication of labour and delivery). Low birthweight and low Apgar-5 were defined as birthweight <2500 g and Apgar score <7, respectively. Each outcome variable was dichotomised into ‘yes’ or ‘no’.

### Covariates

Parity at the index birth (0, 1 and ⩾2), maternal age (<20, 20–24, 25–29, 30–34 and ⩾35 years), socioeconomic status (SES), area of residence (metropolitan, inner region, outer regional, remote and very remote), marital status (never married, married and other), year of birth (1990–1997, 1998–2005 and 2006–2015), pre-existing diabetes (yes/no), hypertension (yes/no) and asthma (yes/no) were included as covariates. Area of residence was determined based on the Australian Statistical Geography Standard remoteness classification (Australian Bureau of Statistics [ABS], 2011), which divides Australia into broad regions that share common characteristics of remoteness for statistical use. Area-based SES disadvantage was derived based on the mother’s place of residence at the time of the index birth and measured using the Australian Bureau of Statistics Index of Relative Socioeconomic Disadvantage (IRSD) from the Census closest to the birth (the adjacent Census was considered when the earlier was not available). The IRSD ranks the relative level of disadvantage of areas using the attributes of all persons in each geographical area and includes measures of income, educational attainment, employment status, occupational skill and housing. Quintiles, first (most disadvantaged) to fifth (least disadvantaged), were determined based on the Australian distribution of the small area values (Pink, 2013). While we used the HMDC and MNS datasets to define pre-existing medical conditions (diabetes, hypertension and asthma), the latter was used to identify pregnancy complications (gestational diabetes, pre-eclampsia and antepartum haemorrhage).

### Statistical analysis

We used descriptive statistics such as proportions to summarise the maternal, perinatal and neonatal characteristics and their distributions across maternal mental health status. A log-binomial (or -Poisson when the earlier failed to converge) model with clustered standard errors (accounting for more than one delivery per woman during the study period) was used to examine the association between maternal mental health disorders and adverse perinatal outcomes. We ran a separate model for each maternal mental health disorder and perinatal outcome, stratified by timing of exposure (in 1 or 5 years preceding birth). Risk ratios (RRs) and 95% confidence intervals (CIs) were calculated using births to women without a mental health disorder as a reference group. We used multinomial logistic regression with clustered standard errors to examine the association between maternal mental disorders and term-SGA, preterm-AGA and preterm-SGA (compared to term-AGA) and obtained RRs and 95% CIs. Each model was adjusted for *a priori* identified and available covariates (year of birth, parity, maternal age, area-based SES, area of residence, marital status, pre-existing diabetes, hypertension and asthma). Pregnancy complications were not included in the models as they were likely on the causal pathway (i.e. intermediate variables) between maternal mental disorders and perinatal outcomes. For example, maternal mental health problems, particularly schizophrenia, were found to be associated with gestational diabetes, which can increase the risk of several adverse perinatal outcomes, including preterm birth and perinatal death (Wilson et al., 2022). Thus, adjustment for gestational diabetes may underestimate the total causal effect of maternal mental disorders on perinatal outcomes.

We repeated the perinatal death models after excluding infant deaths (*n* = 69) reported in the month immediately following the birth month as these could occur beyond the neonatal period. We had 3631 births (8.2%) missing data on any key outcome variables (mainly on 5 minute Apgar score and gestational age) and 2078 (4.7%) on any covariates (notably on the area of residence). Births to women with missing data were less likely to have foetal distress, be born preterm, or be born to nulliparous and first SES quintile (most disadvantaged) women. They were more likely born in 2006–2015 and SGA (Supplemental Table S2). To account for item-level missingness, we used the Multivariate Imputation by Chained Equations (MICE) imputation method with 50 iterations. We ran two separate imputations – for only covariates and all variables (including outcome variables) with missing data. All covariates used in the above regression-based analyses were included in the imputation models. The imputation was conducted in Stata using the ‘mi’ command. Finally, we repeated the main analyses with the imputed datasets and a sample restricted to one randomly selected birth to women. All statistical analyses were performed using STATA version 17 (StataCorp., 2021, College Station, TX, USA).

Ethics approval for the study was obtained from the Western Australian Department of Health Human Ethics Research Committee (RGS0000002808), Murdoch University Human Research Ethics Committee (2021/062) and the Western Australian Aboriginal Health Ethics Committee (983). This work has been conducted as part of the Ngangk Yira Institute for Change (Murdoch University’s Aboriginal health-focused Research Institute, established and led by Aboriginal researchers) Maternal and Child Health Pillar and followed the consolidated criteria for strengthening reporting of health research involving Indigenous peoples (CONSIDER Statement) (Huria et al., 2019). We obtained endorsement from the Institute’s Elders Council. The senior author (Professor Rhonda Marriott) is a highly respected and experienced Aboriginal researcher who provided leadership throughout the project.

## Results

During the study period (1990–2015), there were 45,211 Aboriginal births in WA. After excluding multiple births (*n* = 1044) and births with missing data on outcome variables and covariates (*n* = 5575), 38,592 births from 17,177 mothers were available for all analyses except for low Apgar-5, which had a smaller sample (*n* = 38,458) due to further missing data on this variable.

Nearly a quarter of births (24.2%) were to women under 20 years, about 30% were to nulliparous women, and over half (54.5%) were to most disadvantaged women according to their area-based socioeconomic measure. The proportions of preterm birth, SGA and perinatal death were 14%, 17% and 2%, respectively (Table 1). About 19% of births were to women with at least one mental health disorder reported in 5 years preceding the birth, with approximately 9% reported in the year preceding the birth. Substance use and common mental disorders were the most prevalent mental health disorders reported in 1 and 5 years preceding the birth (see Supplemental Figure S1 for the details).

**Table 1.** Maternal and perinatal characteristics of births to Aboriginal women by maternal mental health status (*N* *=* 38,592), Western Australia.

	Overall	Any mental disorder within 5 years before birth		Any mental disorder within 1 year before birth	
	*N* (%)	No (%)	Yes (%)	No (%)	Yes (%)
Maternal characteristics					
Age in years					
<20	9341 (24.2)	7980 (25.6)	1361 (18.5)	8694 (24.7)	647 (18.8)
20–24	12,978 (33.6)	10,632 (34.0)	2346 (31.9)	11,901 (33.9)	1077 (31.3)
25–29	9152 (23.7)	7199 (23.1)	1953 (26.5)	8233 (23.4)	919 (26.7)
30–34	4856 (12.6)	3698 (11.8)	1158 (15.7)	4320 (12.3)	536 (15.6)
35+	2265 (5.9)	1723 (5.5)	542 (7.4)	2004 (5.7)	261 (7.6)
Parity					
0	11,414 (29.6)	9764 (31.3)	1650 (22.4)	10,588 (30.1)	826 (24.0)
1	9218 (23.9)	7609 (24.4)	1609 (21.9)	8510 (24.2)	708 (20.6)
2+	17,960 (46.5)	13,859 (44.4)	4101 (55.7)	16,054 (45.7)	1906 (55.4)
Marital status					
Never married	12,391 (32.1)	9773 (31.3)	2618 (35.6)	11,025 (31.4)	1366 (39.7)
Married	25,019 (64.8)	20,661 (66.0)	4408 (59.9)	23,109 (65.7)	1910 (55.5)
Other	1182 (3.1)	848 (2.7)	334 (4.5)	1018 (2.9)	164 (4.8)
SES quintiles					
1st (most disadvantaged)	21,020 (54.5)	16,928 (54.2)	4092 (55.6)	19,089 (54.3)	1931 (56.1)
2nd	8639 (22.4)	7047 (22.6)	1592 (21.6)	7910 (22.5)	729 (21.2)
3rd	5457 (14.1)	4416 (14.2)	1041 (14.1)	4964 (14.1)	493 (14.3)
4th	2657 (6.9)	2170 (7.0)	487 (6.6)	2446 (7.0)	211 (6.1)
5th (least disadvantaged)	819 (2.1)	671 (2.1)	148 (2.0)	743 (2.1)	76 (2.2)
Area of residence					
Metropolitan	8163 (21.2)	6289 (20.1)	1874 (25.5)	7187 (20.4)	976 (28.4)
Inner region	7797 (20.2)	6224 (19.9)	1573 (21.4)	7028 (20.0)	769 (22.4)
Outer regional	3684 (9.5)	2898 (9.3)	786 (10.7)	3350 (9.5)	334 (9.7)
Remote	3124 (8.1)	2559 (8.2)	565 (7.7)	2874 (8.2)	250 (7.3)
Very remote	15,824 (41.0)	13,262 (42.5)	2562 (34.8)	14,713 (41.9)	1111 (32.3)
Any pre-existing diabetes	973 (2.5)	662 (2.1)	311 (4.2)	818 (2.3)	155 (4.5)
Pre-existing hypertension	427 (1.1)	315 (1.0)	112 (1.5)	374 (1.1)	53 (1.5)
Pre-existing asthma	5235 (13.6)	3881 (12.4)	1354 (18.4)	4585 (13.0)	650 (18.9)
Perinatal characteristics					
Gestational diabetes	1355 (3.5)	1105 (3.5)	250 (3.4)	1237 (3.5)	118 (3.4)
Pre-eclampsia	1947 (5.1)	1604 (5.1)	343 (4.7)	1781 (5.1)	166 (4.8)
ntepartum haemorrhage	1,070 (2.8)	786 (2.5)	284 (3.9)	918 (2.6)	152 (4.4)
Year of birth					
1990–1997	12,267 (31.8)	10,322 (33.0)	1945 (26.4)	11,400 (32.4)	867 (25.2)
1998–2005	12,962 (33.6)	10,171 (32.6)	2791 (37.9)	11,623 (33.1)	1339 (38.9)
2006–2015	13,363 (34.6)	10,739 (34.4)	2624(35.7)	12,129 (34.5)	1234 (35.9)
Preterm birth	5419 (14.0)	3968 (12.7)	1451 (19.6)	4648 (13.2)	771 (22.4)
Small for gestational age^a^	6594 (17.1)	4900 (15.7)	1694 (23.0)	5736 (16.3)	858 (24.9)
Perinatal death^b^	766 (2.0)	570 (1.8)	196 (2.7)	652 (1.9)	114 (3.3)
Low birthweight (<2500 g)	4825 (12.5)	3351 (10.7)	1474 (19.7)	4003 (11.4)	822 (23.9)
Major birth defect	1701 (4.4)	1267 (4.1)	434 (5.9)	1464 (4.2)	237 (6.9)
Foetal distress	5774 (15.0)	4556 (14.6)	1218 (16.5)	5143 (14.6)	631 (18.3)
Apgar score (<7) at 5 minutes^c^	1414 (3.7)	1077 (3.5)	337 (4.6)	1231 (3.5)	183 (5.3)

SES: socioeconomic status.

a Birthweight < 10^th^ centile for sex and gestation.

b Included 69 infant deaths than could have occurred up to the end of the second months of life as we did not have the exact date of birth data.

c Total sample was smaller (*n* = 38,458) due to missing data.

There were distinct differences in the distribution of most demographic and perinatal characteristics across any maternal mental health disorder (reported in 1 or 5 years preceding the birth). Notably, the proportion of births to multiparous (⩾2), women living in the metropolitan area (Perth) and women with any pre-existing diabetes or asthma were higher for women with any mental health disorder in 1 or 5 years preceding the birth. Additionally, women with any mental health disorder (reported in 1 or 5 years preceding the birth) were more likely to have adverse perinatal outcomes such as perinatal death, preterm birth, low birthweight or SGA, but less likely to be younger or live in very remote areas (Table 1).

Table 2 shows the associations between maternal mental disorders (diagnosed in 1 or 5 years preceding the birth) and primary perinatal outcomes. Births to women with any mental health disorder in 5 years preceding the birth had a 44% to 52% increased risk of being preterm, SGA, or dying in the perinatal period, compared with births to women without a history of mental health disorders. By category, the maternal mental health disorders that were consistently associated with these perinatal outcomes included severe mental disorders, common mental disorders and substance use disorders. We found similar but slightly stronger associations for a diagnosis of a mental health disorder reported in the year preceding the birth and the primary perinatal outcomes, with a statistically significant association between maternal personality disorders and preterm birth.

**Table 2.** The associations between maternal mental health disorders and perinatal (primary) outcomes among births to Aboriginal women (*N* *=* 38,592), Western Australia.

Women with:	Row N	Preterm birth		Small for gestational age^a^		Perinatal death^b^	
		Cases	aRR [95% CI]	Cases	aRR [95% CI]	Cases	aRR [95% CI]
Within 5 years before birth							
Any mental disorder	7360	1451	1.48 [1.39, 1.57]	1694	1.52 [1.45, 1.61]	196	1.44 [1.22, 1.70]
Severe mental disorder	585	112	1.41 [1.17, 1.70]	119	1.37 [1.15, 1.64]	14	1.29 [0.77, 2.17]
Common mental disorder	3355	631	1.43 [1.31, 1.55]	649	1.29 [1.19, 1.40]	85	1.43 [1.13, 1.80]
Personality disorder	245	44	1.31 [0.94, 1.82]	45	1.21 [0.93, 1.56]	<10	1.09 [0.45, 2.64]
Substance use disorder	4567	966	1.57 [1.46, 1.69]	1210	1.77 [1.67, 1.88]	123	1.42 [1.16, 1.74]
Other adult-onset disorder	1420	238	1.26 [1.11, 1.43]	329	1.49 [1.34, 1.66]	36	1.36 [0.97, 1.91]
Childhood-onset disorder	165	27	1.20 [0.84, 1.70]	42	1.47 [1.12, 1.92]	<10	1.88 [0.85, 4.18]
No mental disorder	31,232	3968	1.00	4900	1.00	570	1.00
Within 1 year before birth							
Any mental disorder	3440	771	1.59 [1.48, 1.71]	858	1.57 [1.47, 1.67]	114	1.74 [1.42, 2.13]
Severe mental disorder	242	56	1.58 [1.25, 2.00]	53	1.39 [1.09, 1.76]	10	2.11 [1.15, 3.86]
Common mental disorder	1397	298	1.54 [1.38, 1.72]	261	1.18 [1.05, 1.32]	49	1.93 [1.44, 2.58]
Personality disorder	87	21	1.62 [1.07, 2.45]	15	1.07 [0.67, 1.66]	<10	1.84 [0.60, 5.70]
Substance use disorder	2103	505	1.69 [1.55, 1.84]	632	1.89 [1.76, 2.03]	61	1.47 [1.13, 1.92]
Other adult-onset disorder	298	57	1.31 [1.04, 1.67]	49	1.01 [0.77, 1.32]	10	1.70 [0.92, 3.16]
Childhood-onset disorder	43	<10	1.12 [0.57, 2.19]	14	1.78 [1.16, 2.73]	<10	2.24 [0.58, 8.58]
No mental disorder	35,152	4648	1.00	5736	1.00	652	1.00

aRR: adjusted relative risk; CI: confidence interval.

Models were adjusted for socio-demographic characteristics (parity, maternal age, area-based socioeconomic status, area of residence, marital status and year of birth) and pre-existing conditions (asthma, diabetes and hypertension).

a Birthweight < 10^th^ centile for sex and gestation.

b Included 69 infant deaths than could have occurred up to the end of the second months of life as we did not have the exact date of birth data.

Births to women with any mental disorder (in 5 years preceding the birth) were more likely to be preterm-SGA (RR = 2.63, 95% CI = [2.21, 3.13]) than term-AGA relative to births to women without a mental disorder. They were also more likely to be preterm-AGA (RR = 1.69, 95% CI = [1.56, 1.83]) or term-SGA (RR = 1.78, 95% CI = [1.65, 1.91]) than term-AGA relative to babies born to women with no mental disorder. We found similar association patterns across maternal mental disorder categories (reported in 1 or 5 years preceding the birth) and stronger associations for maternal mental disorders occurring in one year preceding the birth and preterm-SGA. The strongest association was found between maternal substance use disorder (reported in the year preceding the birth) and preterm-SGA (RR = 4.20, 95% CI = [3.32, 5.31]) (Table 3).

**Table 3.** The associations between maternal mental disorders and preterm and SGA categories among births to Aboriginal women (*N* *=* *38, 592*), Western Australia.

Women with:	aRR [95% CI]			
	Term-AGA	Preterm-SGA	Preterm-AGA	Term-SGA
Within 5 years before birth				
Any mental disorder	1.00	2.63 [2.21, 3.13]	1.69 [1.56, 1.83]	1.78 [1.65, 1.91]
Severe mental disorder	1.00	2.15 [1.26, 3.68]	1.58 [1.23, 2.04]	1.53 [1.20, 1.93]
Common mental disorder	1.00	2.23 [1.74, 2.86]	1.55 [1.38, 1.74]	1.39 [1.24, 1.55]
Personality disorder	1.00	0.81 [0.25, 2.59]	1.56 [1.01, 2.42]	1.42 [1.02, 1.99]
Substance use disorder	1.00	3.20 [2.62, 3.92]	1.93 [1.74, 2.13]	2.24 [2.05, 2.44]
Other adult-onset disorder	1.00	2.44 [1.74, 3.42]	1.33 [1.12, 1.59]	1.65 [1.42, 1.92]
Childhood-onset disorder	1.00	2.54 [1.10, 5.87]	1.22 [0.74, 2.02]	1.60 [1.07, 2.39]
No mental disorder	1.00	1.00	1.00	1.00
Within 1 year before birth				
Any mental disorder	1.00	3.14 [2.55, 3.85]	1.89 [1.71, 2.09]	1.86 [1.69, 2.05]
Severe mental disorder	1.00	2.50 [1.25, 5.03]	1.90 [1.35, 2.67]	1.59 [1.12, 2.25]
Common mental disorder	1.00	2.23 [1.61, 3.09]	1.70 [1.46, 1.98]	1.24 [1.06, 1.45]
Personality disorder	1.00	2.15 [0.65, 7.09]	1.83 [1.00, 3.35]	1.08 [0.58, 2.02]
Substance use disorder	1.00	4.20 [3.32, 5.31]	2.17 [1.91, 2.47]	2.51 [2.24, 2.81]
Other adult-onset disorder	1.00	1.69 [0.82, 3.50]	1.36 [0.98, 1.89]	0.99 [0.70, 1.40]
Childhood-onset disorder	1.00	2.95 [0.69, 12.65]	1.15 [0.42, 3.12]	2.14 [1.06, 4.32]
No mental disorder	1.00	1.00	1.00	1.00

SGA: small for gestational age (birthweight < 10th centile for sex and gestation); aRR: adjusted relative risk; CI: confidence interval; AGA: appropriate for gestational age. Models were adjusted for socio-demographic characteristics (parity, maternal age, area-based socioeconomic status, area of residence, marital status and year of birth) and pre-existing conditions (asthma, diabetes and hypertension).

We also found associations between any maternal mental health disorders (reported in 1 or 5 years preceding the birth) and all secondary perinatal outcomes (major congenital anomalies, low birthweight, low Apgar score at 5 minutes and foetal distress), although the strength of the association varied across the outcomes (RRs ranged from 1.18 to 2.00) and was consistently elevated for any mental health disorders reported in the year preceding the birth and low birthweight (Table 4). While substance use disorder (RRs ranged from 1.25 to 2.34) and common mental disorder (RRs ranged from 1.14 to 1.61) were significantly associated with all secondary perinatal outcomes, the other types of maternal mental health disorders apart from childhood-onset disorder were significantly associated with at least one perinatal outcome.

**Table 4.** The associations between maternal mental health disorders and perinatal (secondary) outcomes among births to Aboriginal women (*N* *=* 38,592), Western Australia.

Women with:	Row N	Major birth defect		Low birthweight		Low Apgar-5^a^		Foetal distress	
		Cases	aRR [95% CI]	Cases	aRR [95% CI]	Cases	aRR [95% CI]	Cases	aRR [95% CI]
Within 5 years before birth									
Any mental disorder	7360	434	1.41 [1.27, 1.58]	1474	1.81 [1.70, 1.93]	337	1.35 [1.19, 1.53]	1218	1.18 [1.11, 1.25]
Severe mental disorder	585	31	1.24 [0.87, 1.77]	107	1.64 [1.33, 2.01]	28	1.41 [0.97, 2.07]	86	1.06 [0.86, 1.29]
Common mental disorder	3355	167	1.21 [1.03, 1.43]	589	1.60 [1.46, 1.75]	146	1.33 [1.11, 1.59]	522	1.14 [1.04, 1.24]
Personality disorder	245	16	1.54 [0.91, 2.60]	38	1.34 [0.95, 1.89]	12	1.42 [0.73, 2.75]	45	1.19 [0.92, 1.54]
Substance use disorder	4567	314	1.62 [1.43, 1.84]	1054	2.08 [1.94, 2.24]	212	1.36 [1.18, 1.59]	800	1.25 [1.17, 1.35]
Other adult-onset disorder	1420	75	1.29 [1.01, 1.62]	239	1.52 [1.34, 1.74]	60	1.22 [0.93, 1.59]	236	1.12 [0.99, 1.27]
Childhood-onset disorder	165	<10	1.31 [0.70, 2.44]	26	1.34 [0.94, 1.92]	<10	1.26 [0.65, 2.46]	32	1.06 [0.79, 1.43]
No mental disorder	31,232	1267	1.00	3351	1.00	1077	1.00	4556	1.00
Within 1 year before birth									
Any mental disorder	3440	237	1.60 [1.39, 1.83]	822	2.00 [1.87, 2.15]	183	1.51 [1.29, 1.77]	631	1.26 [1.17, 1.36]
Severe mental disorder	242	13	1.19 [0.70, 2.02]	56	1.86 [1.47, 2.35]	19	2.20 [1.42, 3.42]	46	1.30 [0.99, 1.71]
Common mental disorder	1397	83	1.39 [1.12, 1.73]	268	1.61 [1.44, 1.81]	75	1.58 [1.25, 1.99]	229	1.15 [1.02, 1.30]
Personality disorder	87	<10	1.55 [0.71, 3.38]	15	1.36 [0.85, 2.17]	<10	1.92 [0.88, 4.15]	22	1.54 [1.09, 2.19]
Substance use disorder	2103	172	1.87 [1.59, 2.19]	589	2.34 [2.16, 2.53]	101	1.35 [1.10, 1.66]	415	1.34 [1.22, 1.47]
Other adult-onset disorder	298	24	1.83 [1.24, 2.70]	54	1.48 [1.16, 1.90]	15	1.38 [0.84, 2.27]	39	0.90 [0.67, 1.22]
Childhood-onset disorder	43	<10	0.51 [0.07, 3.53]	<10	1.27 [0.64, 2.50]	<10	1.14 [0.31, 4.16]	10	1.16 [0.69, 1.96]
No mental disorder	35,152	1464	1.00	4003	1.00	1231	1.00	5143	1.00

aRR: adjusted relative risk; CI: confidence interval.

Models were adjusted for socio-demographic characteristics (parity, maternal age, area-based socioeconomic status, area of residence, marital status and year of birth) and pre-existing conditions (asthma, diabetes and hypertension).

a Total sample was smaller (*n* = 38,458) due to missing data and the Raw N may not be applicable.

Overall, there were no meaningful changes in effect estimates (RRs) when the analyses were repeated with imputed datasets (Supplemental Tables S3 and S4) or after excluding infant deaths reported in the month immediately following the birth month (data not shown). The results of additional analyses (restricted to one birth per woman) were not substantially different from the primary analysis results, although some of the estimates were less precise (Supplemental Tables S5 and S6). We also found similar results when using a binary outcome (yes/no) variable that captured any of the adverse perinatal outcomes of interest (Table S7).

## Discussion

In this population-based cohort study, we found statistically significant associations between most types of maternal mental health disorders and a range of adverse perinatal outcomes (preterm birth, SGA, perinatal death, major congenital anomalies, foetal distress, low birthweight and low Apgar score) among babies born to Aboriginal women. Overall, the associations did not vary substantially by the types of maternal mental disorder and perinatal outcomes, although they were slightly stronger in cases where the maternal mental health contact was proximal to the birth (i.e. diagnosed in the year preceding the birth) and for substance use disorder. Additionally, our study demonstrated consistently stronger associations for almost all maternal mental disorders and preterm births with poor foetal growth compared to births with other gestational age and growth combinations, providing additional evidence for the causal claim (Lewis et al., 2015).

The impacts of depression and anxiety problems during pregnancy on perinatal outcomes, particularly on low birthweight and preterm birth, have been well studied in the general population. Our effect estimates for the associations between common mental disorders (including depression and anxiety) and low birthweight and preterm birth are comparable with the extant findings among studies of mainstream populations (Ding et al., 2014; Ghimire et al., 2021; Grigoriadis et al., 2018). However, this is the first comprehensive study to show the effect of maternal mental health disorders across the diagnosis spectrum for the Australian Aboriginal population – thereby expanding from the existing limited evidence focusing only on substance use disorders (Adane et al., 2022). These findings broadly underscore the need for further research (to establish the evidence base and elucidate the underlying mechanisms) and suggest that targeted perinatal mental health interventions that provide appropriate support to Aboriginal mothers will improve perinatal outcomes.

We also demonstrated a heightened risk of perinatal mortality for women with mental health disorders (including severe, common and substance use disorders), particularly when Aboriginal women had these conditions reported around the time of pregnancy (in the year before birth). These findings extended the detrimental effect of poor maternal mental health beyond early life morbidity outcomes and are consistent with the results of our previous systematic review and meta-analysis (Adane et al., 2021), which included 28 studies. In contrast to our study, a recent study in the state of Victoria, Australia, did not find significant associations between severe maternal mental illness and stillbirth or neonatal death (Edvardsson et al., 2022). This could be due to variations in the study population and control group. While the current study included only singleton births to Aboriginal women and used women without any mental health disorders as a reference group, the Victorian study included all singleton births to Aboriginal and non-Aboriginal women. The latter also used women without severe mental illness as a control group, which could involve women with other mental health disorders. Our findings on SGA, congenital anomalies, low 5-minute Apgar score and foetal distress are consistent with the previous literature showing associations between severe maternal mental illness and foetal distress, anomalies or low Apgar score (Edvardsson et al., 2022; Etchecopar-Etchart et al., 2022; Heun-Johnson et al., 2019; Zhong et al., 2018); and between major depression, bipolar or substance use disorder and SGA (Mei-Dan et al., 2015; O’Leary et al., 2020). However, investigations of this nature are rare in Australian Aboriginal populations, suggesting the need for more well-designed prospective studies to replicate our finding and strengthen the evidence base. What cannot be elucidated in the current data is whether Aboriginal women who are seen by public mental health services are more likely to be given a mental health diagnosis and what cultural and contextual considerations were made in this process. Furthermore, the extent to which Aboriginal women who present with mental health disorders receive evidence-informed interventions and treatment is unknown. Given the plethora of literature describing the impact of racism and unconscious bias within health services, including mental health services, on access to appropriate services, diagnosis and treatment, it is likely this is an unaccounted-for factor in our examination of both mental health and perinatal outcomes (Kelaher et al., 2014; Peek, 2021; Talamaivao et al., 2020).

A recent systematic review and meta-analysis (including five studies of 1114 women with a personality disorder and 8,527,780 control women) reported a greater risk of preterm birth, low birthweight and low Apgar score (pooled ORs ranging from 2.0 to 2.6) for women with a personality disorder (Marshall et al., 2020). In line with this, we showed an association between maternal personality disorder and some perinatal outcomes (preterm birth and foetal distress). But the association with our other key perinatal outcomes, including low birthweight and Apgar score, did not reach statistical significance – perhaps due to the small sample sizes.

Our analysis included all WA Aboriginal singleton births during the study period, which minimised selection and response biases, provided statistical power to evaluate relatively rare perinatal outcomes (e.g. perinatal death) and enhanced the generalisability of our findings. Unlike most previous studies, we have comprehensively assessed the full spectrum of maternal mental health disorders based on standard assessment and classification. The study also had some limitations. We used administrative data, which is prone to misclassification and other errors. Our mental health data sources (HMDC and MHIS) may not capture all mental health cases, particularly less severe cases that did not result in a diagnosis as well as women who sought mental health care through their general practitioner, Aboriginal Medical Service and private practitioners including private psychologists and psychiatrists. Further, these diagnoses are based on clinical records and assessments that are then entered into the database not diagnostic interviews and validated measures of mental health diagnoses. This means women with mental health disorders could be misclassified and reduce the strength of the association between maternal mental disorders and perinatal outcomes. Although we have adjusted for a range of factors, we have no data on smoking during pregnancy, alcohol consumption, obesity, pharmacological treatments in pregnancy for mental health disorders and the level of antenatal and mental health care, which are major issues for individuals with mental health disorders (Ben-Sheetrit et al., 2018; Scott and Happell, 2011). We lack data on whether the preterm birth is due to spontaneous labour or medically indicated and statistical power to examine the dose–response relationship (if any) between maternal mental health disorders and the severity of preterm birth. We had limited statistical power for some mental health disorders (e.g. personality disorder) and neonatal outcomes (e.g. perinatal death) and did not reach a definitive conclusion in these cases.

Generally, most of the study effect sizes are in the small to moderate range and likely, at least partly, due to the clustering of adverse maternal risk factors among women who experience poor mental health disorders (Gibberd et al., 2019; Vermeulen-Smit et al., 2015). However, given the prevalence of mental health conditions is high in the Aboriginal population in general and in women during their perinatal period (Ogilvie et al., 2021; Owais et al., 2020; Page et al., 2022), these findings have important implications and relevance for public health prevention. Furthermore, what is evident is structural barriers to access to mental health care over the perinatal period that urgently need to be addressed (Bhat et al., 2020; Denison et al., 2014). This includes specific perinatal mental health services such as Mother Baby Units (Galbally et al., 2019). The impact of maternal mental health on preterm birth and low birthweight is of interest as these are critical public health issues in the Aboriginal population (AIHW, 2021) and are well-known indicators of health and development outcomes across the life course (Wolke et al., 2019). Therefore, overall integrated prevention of mental health problems and early identification, holistic, trauma-informed antenatal care, support and treatment for mental health that is also culturally sensitive and safe for Aboriginal women are urgently required to improve women’s health and wellbeing and their pregnancy and neonatal outcomes (Howard and Khalifeh, 2020; Reibel et al., 2015; Reibel and Walker, 2010). Such culturally sensitive wrap-around services (co-designed and community-led) have been shown to improve the rate of antenatal care attendance, preterm birth and breastfeeding among the First Nations Australians (Kildea et al., 2021). Cultural safety requires employing more Aboriginal health and mental health workers, increasing the cultural competency of non-Aboriginal health practitioners and developing health service policies in collaboration with Aboriginal peak bodies and the local communities (Upton et al., 2021).

## Conclusion

In this large population-based retrospective cohort study, we found negative associations between maternal mental disorders across the diagnostic spectrum and perinatal outcomes after adjustment for sociodemographic factors and pre-existing comorbid conditions. The size of the associations did not considerably vary when examined by the types of maternal mental disorders. The effect sizes were of the small to moderate range and were slightly stronger for mental health disorders reported around pregnancy and substance use disorders. Prevention and early identification of Aboriginal women with mental health disorders using culturally validated tools, provision of holistic antenatal care, access to culturally sensitive and safe mental health treatment and support for Aboriginal women may improve adverse perinatal and later life outcomes.

## Supplemental Material

sj-docx-1-anp-10.1177_00048674231160986 – Supplemental material for Perinatal outcomes of Aboriginal women with mental health disorders Click here for additional data file. Supplemental material, sj-docx-1-anp-10.1177_00048674231160986 for Perinatal outcomes of Aboriginal women with mental health disorders by Akilew A Adane, Carrington CJ Shepherd, Roz Walker, Helen D Bailey, Megan Galbally and Rhonda Marriott in Australian & New Zealand Journal of Psychiatry
