# Supplementary material for: Perinatal outcomes of Aboriginal women with mental health disorders
Source: Aust N Z J Psychiatry. 2023 Mar 16;57(10):1331–42. doi: 10.1177/00048674231160986 (PMC10517592; doi:10.1177/00048674231160986)
Supplement: sj-docx-1-anp-10.1177_00048674231160986 – Supplemental material for Perinatal outcomes of Aboriginal women with mental health disorders [file sj-docx-1-anp-10.1177_00048674231160986.docx]

**Supplement to ‘Perinatal outcomes of Aboriginal women with mental health disorders’**

**By Authors**

Akilew A Adane, Carrington CJ Shepherd, Roz Walker, Helen D Bailey, Megan Galbally, Rhonda Marriott

Table S1. Broad maternal mental illness diagnostic category definitions^a^

| Broad Diagnostic Group | Diagnostic categories | ICD-10 codes | ICD-9 codes |
| --- | --- | --- | --- |
| Severe mental disorder | Schizophrenia and other psychotic disorders | F20, F21, F22, F23, F24, F28, F29 | 295-298 |
|  | Schizoaffective Disorders | F25 |  |
|  | Psychotic Affective Disorders | F30, F31 |  |
|  | Psychotic Disorders related to Substance Use | F10.5, F11.5, F12.5, F13.5, F14.5, F15.5, F15.50, F15.51, F15.59, F15.70, F16.5, F17.5, F18.5, F19.5, F19.7 | 291.3, 291.5, 292.1 |
| Common mental disorder | Non-psychotic Affective Disorders | F32, F33, F34, F38, F39, F53 | 300, 306, 308, 309, 311 |
|  | Neurotic and stress-related (anxiety) disorders | F40, F41, F42, F43, F44, F45, F48 |  |
| Personality disorder | Cluster A | F60.0, F60.1 | 301 |
|  | Cluster B | F60.2, F60.3, F60.30, F60.31, F60.4 |  |
|  | Cluster C | F60.5, F60.6, F60.7 |  |
|  | Other Personality Disorders | F60, F60.8, F60.9, F61, F62, F68, F69 |  |
| Substance use disorder | Substance Intoxication and Use Disorders | F10, F11, F12, F13, F14, F15, F16, F17, F18, F19 | 291, 292, 303, 304, 305 |
| Other adult-onset disorder | Other Disorders not elsewhere classified | F99 | 290, 293, 294, 302, 307, 310, 316  E950-E959  299, 312-315, 317-319 |
|  | Other Adult-Onset Disorders (e.g., organic disorders, eating disorders, sleep disorders, sexual, disorders, and abuse of non-dependence-producing substances) | F00, F01, F03, F04, F05, F06, F07, F09, F50, F51, F52, F54, F55, F59, F63, F64, F65, F66 |  |
|  | Self-harm and suicidal ideation | R45.81, X60-X84 |  |
| Childhood-onset disorder | Hyperkinetic disorders | F90 |  |
|  | Conduct Disorders | F91, F92 |  |
|  | Other Childhood Disorders (e.g., hyperkinetic disorders, conduct disorders, mental retardation, pervasive developmental disorders) | F70, F71, F72, F73, F78, F79, F80, F81, F82, F83, F84, F88, F89, F93, F94, F95, F98 |  |

*^a^Only Broad Diagnostic Groups were used in the analysis.*

Fig S1. Proportion of births to Aboriginal mothers with a mental disorder, by category and timing of contact, Western Australia, 1990-2015

Table S2. Maternal and perinatal characteristics of births to Aboriginal women by missing data status (N = 44,167), Western Australia

|  | Missing data | | | |
| --- | --- | --- | --- | --- |
| Characteristics | No | | Yes | |
| Age in years | N | % | N | % |
| <20 | 9,309 | 24.2 | 1,202 | 21.1 |
| 20-24 | 12,942 | 33.7 | 1,946 | 34.1 |
| 25-29 | 9,113 | 23.7 | 1,375 | 24.1 |
| 30-34 | 4,839 | 12.6 | 729 | 12.8 |
| 35+ | 2,255 | 5.9 | 454 | 8.0 |
| Parity |  |  |  |  |
| 0 | 11,392 | 29.6 | 732 | 17.1 |
| 1 | 9,182 | 23.9 | 1,293 | 30.2 |
| 2 | 17,884 | 46.5 | 2,262 | 52.8 |
| Marital status |  |  |  |  |
| Never married | 12,337 | 32.1 | 700 | 31.4 |
| Married | 24,942 | 64.9 | 1,476 | 66.2 |
| Other | 1,179 | 3.1 | 54 | 2.4 |
| SES quintiles |  |  |  |  |
| 1^st^ (most disadvantaged) | 20,936 | 54.4 | 859 | 39.1 |
| 2^nd^ | 8,609 | 22.4 | 539 | 24.6 |
| 3^rd^ | 5,441 | 14.1 | 544 | 24.8 |
| 4^th^ | 2,653 | 6.9 | 204 | 9.3 |
| 5^th^ (least disadvantaged) | 819 | 2.1 | 49 | 2.2 |
| Area of residence |  |  |  |  |
| Metropolitan | 8,138 | 21.2 | 28 | 18.2 |
| Inner region | 7,773 | 20.2 | 38 | 24.7 |
| Outer regional | 3,677 | 9.6 | <10 | 5.2 |
| Remote | 3,114 | 8.1 | 12 | 7.8 |
| Very remote | 15,756 | 41.0 | 68 | 44.2 |
| Any pre-existing diabetes |  |  |  |  |
| Yes | 972 | 2.5 | 180 | 3.2 |
| Pre-existing hypertension |  |  |  |  |
| Yes | 426 | 1.1 | 54 | 0.9 |
| Pre-existing asthma |  |  |  |  |
| Yes | 5,225 | 13.6 | 579 | 10.1 |
| Year of birth |  |  |  |  |
| 1990-1997 | 12,208 | 31.7 | 776 | 13.6 |
| 1998-2005 | 12,911 | 33.6 | 1,149 | 20.1 |
| 2006-2015 | 13,339 | 34.7 | 3,784 | 66.3 |
| Preterm birth |  |  |  |  |
| Yes | 5,366 | 14.0 | 533 | 9.3 |
| Low birthweight (<2500g) |  |  |  |  |
| Yes | 4,775 | 12.4 | 760 | 13.3 |
| Major birth defect |  |  |  |  |
| Yes | 1,689 | 4.4 | 236 | 4.1 |
| Small for gestational age |  |  |  |  |
| Yes | 6,555 | 17.0 | 508 | 21.5 |
| Perinatal death |  |  |  |  |
| Yes | 757 | 2.0 | 146 | 2.6 |
| Fetal distress |  |  |  |  |
| Yes | 5,768 | 15.0 | 311 | 5.4 |
| Apgar score (<7) at 5 minutes |  |  |  |  |
| Yes | 1,414 | 3.7 | 91 | 4.4 |
| *Within five years before birth* |  |  |  |  |
| Any mental disorders |  |  |  |  |
| Yes | 7,325 | 19.0 | 1,113 | 19.5 |
| Severe mental disorder |  |  |  |  |
| Yes | 581 | 1.8 | 101 | 2.2 |
| Common mental disorder |  |  |  |  |
| Yes | 3,345 | 9.7 | 514 | 10.1 |
| Personality disorder |  |  |  |  |
| Yes | 244 | 0.8 | 61 | 1.3 |
| Substance use disorder |  |  |  |  |
| Yes | 4,543 | 12.7 | 755 | 14.1 |
| Other adult-onset disorder |  |  |  |  |
| Yes | 1,411 | 4.3 | 228 | 4.7 |
| Childhood-onset disorder |  |  |  |  |
| Yes | 163 | 0.5 | 25 | 0.5 |
| *Within one year before birth* |  |  |  |  |
| Any mental disorders |  |  |  |  |
| Yes | 3,426 | 8.9 | 466 | 8.2 |
| Severe mental disorder |  |  |  |  |
| Yes | 241 | 0.7 | 57 | 1.1 |
| Common mental disorder |  |  |  |  |
| Yes | 1,392 | 3.8 | 201 | 3.7 |
| Personality disorder |  |  |  |  |
| Yes | 86 | 0.2 | 20 | 0.4 |
| Substance use disorder |  |  |  |  |
| Yes | 2,095 | 5.6 | 307 | 5.5 |
| Other adult-onset disorder |  |  |  |  |
| Yes | 297 | 0.8 | 50 | 0.9 |
| Childhood-onset disorder |  |  |  |  |
| Yes | 41 | 0.1 | <10 | 0.1 |

Table S3. The associations between maternal mental disorders and perinatal outcomes among birth to Aboriginal women: imputed covariate with missing data (*N = 40,536*), Western Australia

|  | aRR (95% CI) | | | | | | |
| --- | --- | --- | --- | --- | --- | --- | --- |
| Women with: | Preterm birth | SGA | Perinatal death | Major birth defect | Low birthweight (<2500g) | Low Apgar score (<7) at 5 minutes | Fetal distress |
| *Within 5 years before birth* |  |  |  |  |  |  |  |
| Any mental disorder | 1.46 (1.37, 1.55) | 1.53 (1.46, 1.62) | 1.36 (1.16, 1.61) | 1.44 (1.30, 1.61) | 1.80 (1.69, 1.91) | 1.34 (1.18, 1.51) | 1.17 (1.10, 1.24) |
| Severe mental disorder | 1.39 (1.16, 1.67) | 1.44 (1.21, 1.69) | 1.11 (0.65, 1.91) | 1.24 (0.88, 1.76) | 1.60 (1.31, 1.95) | 1.37 (0.95, 1.99) | 1.08 (0.89, 1.31) |
| Common mental disorder | 1.41 (1.30, 1.54) | 1.29 (1.19, 1.40) | 1.38 (1.10, 1.75) | 1.21 (1.03, 1.42) | 1.59 (1.45, 1.74) | 1.31 (1.09, 1.56) | 1.13 (1.03, 1.23) |
| Personality disorder | 1.39 (1.02, 1.88) | 1.16 (0.90, 1.50) | 1.03 (0.43, 2.48) | 1.55 (0.94, 2.57) | 1.40 (1.02, 1.93) | 1.35 (0.69, 2.62) | 1.23 (0.96, 1.58) |
| Substance use disorder | 1.55 (1.44, 1.66) | 1.79 (1.69, 1.90) | 1.35 (1.11, 1.65) | 1.67 (1.48, 1.88) | 2.06 (1.92, 2.21) | 1.35 (1.17, 1.56) | 1.24 (1.16, 1.33) |
| Other adult-onset disorder | 1.25 (1.10, 1.41) | 1.48 (1.33, 1.64) | 1.24 (0.88, 1.74) | 1.33 (1.06, 1.66) | 1.51 (1.33, 1.72) | 1.18 (0.91, 1.54) | 1.10 (0.97, 1.24) |
| Childhood-onset disorder | 1.07 (0.74, 1.54) | 1.47 (1.13, 1.92) | 1.51 (0.63, 3.62) | 1.25 (0.67, 2.33) | 1.24 (0.86, 1.78) | 1.20 (0.61, 2.34) | 1.11 (0.82, 1.49) |
| No mental disorder | 1.00 | 1.00 | 1.00 | 1.00 | 1.00 | 1.00 | 1.00 |
| *Within 1 year before birth* |  |  |  |  |  |  |  |
| Any mental disorder | 1.57 (1.46, 1.68) | 1.59 (1.50, 1.70) | 1.61 (1.31, 1.97) | 1.60 (1.40, 1.83) | 1.99 (1.85, 2.13) | 1.46 (1.25, 1.71) | 1.25 (1.16, 1.35) |
| Severe mental disorder | 1.61 (1.29, 2.01) | 1.45 (1.16, 1.81) | 1.93 (1.05, 3.54) | 1.10 (0.65, 1.87) | 1.86 (1.48, 2.34) | 2.16 (1.41, 3.29) | 1.31 (1.01, 1.71) |
| Common mental disorder | 1.52 (1.37, 1.69) | 1.19 (1.06, 1.33) | 1.82 (1.36, 2.43) | 1.37 (1.11, 1.70) | 1.59 (1.42, 1.78) | 1.51 (1.20, 1.90) | 1.15 (1.02, 1.30) |
| Personality disorder | 1.72 (1.16, 2.54) | 1.01 (0.64, 1.59) | 1.74 (0.56, 5.39) | 1.49 (0.68, 3.23) | 1.47 (0.95, 2.28) | 1.82 (0.84, 3.95) | 1.58 (1.12, 2.23) |
| Substance use disorder | 1.66 (1.52, 1.81) | 1.93 (1.80, 2.07) | 1.38 (1.06, 1.79) | 1.87 (1.60, 2.18) | 2.32 (2.14, 2.51) | 1.31 (1.07, 1.60) | 1.32 (1.21, 1.45) |
| Other adult-onset disorder | 1.34 (1.07, 1.69) | 0.98 (0.75, 1.27) | 1.59 (0.86, 2.95) | 1.75 (1.19, 2.58) | 1.46 (1.17, 1.88) | 1.30 (0.79, 2.13) | 0.85 (0.62, 1.15) |
| Childhood-onset disorder | 0.79 (0.35, 1.77) | 1.99 (1.34, 2.95) | 1.10 (0.16, 7.80) | 0.49 (0.07, 3.43) | 1.07 (0.51, 2.24) | 1.07 (0.29, 3.93) | 1.18 (0.71, 1.98) |
| No mental disorder | 1.00 | 1.00 | 1.00 | 1.00 | 1.00 | 1.00 | 1.00 |

*aRR, adjusted relative risk; SGA, small-for-gestational age (birthweight <10^th^ centile for sex and gestation)*

*Models were adjusted for socio-demographic characteristics (parity, maternal age, area based socioeconomic status, area of residence, marital status, and year of birth) and pre-existing conditions (asthma, diabetes, and hypertension)*

Table S4. The associations between maternal mental disorders and perinatal outcomes among Aboriginal women: imputed all variables with missing data (*N =44,167*), Western Australia

|  | aRR (95% CI) | | | | | | |
| --- | --- | --- | --- | --- | --- | --- | --- |
| Women with: | Preterm birth | SGA | Perinatal death | Major birth defect | Low birthweight (<2500g) | Apgar score (<7) at 5 minutes | Fetal distress |
| *Within 5 years before birth* |  |  |  |  |  |  |  |
| Any mental disorder | 1.47 (1.38, 1.55) | 1.53 (1.45, 1.61) | 1.38 (1.18, 1.61) | 1.44 (1.30, 1.60) | 1.69 (1.69, 1.91) | 1.35 (1.20, 1.52) | 1.18 (1.11, 1.25) |
| Severe mental disorder | 1.44 (1.21, 1.72) | 1.43 (1.22, 1.68) | 1.35 (0.85, 2.14) | 1.21 (0.87, 1.69) | 1.65 (1.37, 2.00) | 1.48 (1.04, 2.11) | 1.10 (0.90, 1.33) |
| Common mental disorder | 1.42 (1.31, 1.55) | 1.30 (1.21, 1.40) | 1.41 (1.13, 1.75) | 1.23 (1.05, 1.43) | 1.58 (1.45, 1.73) | 1.32 (1.11, 1.57) | 1.15 (1.05, 1.25) |
| Personality disorder | 1.29 (0.95, 1.76) | 1.22 (0.96, 1.56) | 0.85 (0.36, 2.06) | 1.68 (1.07, 2.63) | 1.36 (1.00, 1.86) | 1.21 (0.62, 2.36) | 1.21 (0.94, 1.56) |
| Substance use disorder | 1.56 (1.45, 1.67) | 1.76 (1.66, 1.86) | 1.40 (1.16, 1.68) | 1.65 (1.47, 1.86) | 2.05 (1.91, 2.19) | 1.38 (1.19, 1.58) | 1.25 (1.17, 1.34) |
| Other adult-onset disorder | 1.23 (1.09, 1.40) | 1.49 (1.34, 1.64) | 1.22 (0.88, 1.69) | 1.30 (1.05, 1.62) | 1.52 (1.34, 1.71) | 1.17 (0.91, 1.52) | 1.09 (0.96, 1.22) |
| Childhood-onset disorder | 1.10 (0.77, 1.56) | 1.50 (1.17, 1.92) | 1.61 (0.73, 3.57) | 1.42 (0.81, 2.50) | 1.36 (0.97, 1.89) | 1.25 (0.66, 2.36) | 1.08 (0.80, 1.47) |
| No mental disorder | 1.00 | 1.00 | 1.00 | 1.00 | 1.00 | 1.00 | 1.00 |
| *Within 1 year before birth* |  |  |  |  |  |  |  |
| Any mental disorder | 1.59 (1.48, 1.71) | 1.57 (1.47, 1.67) | 1.66 (1.37, 2.01) | 1.61 (1.42, 1.84) | 1.98 (1.85, 2.11) | 1.49 (1.28, 1.73) | 1.27 (1.18, 1.37) |
| Severe mental disorder | 1.63 (1.31, 2.02) | 1.45 (1.16, 1.80) | 2.29 (1.36, 3.86) | 1.13 (0.69, 1.85) | 1.89 (1.53, 2.34) | 2.29 (1.54, 3.40) | 1.30 (1.00, 1.69) |
| Common mental disorder | 1.55 (1.39, 1.72) | 1.19 (1.07, 1.34) | 1.87 (1.43, 2.45) | 1.39 (1.13, 1.72) | 1.59 (1.42, 1.77) | 1.55 (1.24, 1.93) | 1.18 (1.04, 1.33) |
| Personality disorder | 1.62 (1.09, 2.40) | 1.13 (0.75, 1.70) | 1.48 (0.48, 4.51) | 1.53 (0.75, 3.15) | 1.52 (1.00, 2.31) | 1.63 (0.75, 3.53) | 1.57 (1.11, 2.21) |
| Substance use disorder | 1.68 (1.54, 1.82) | 1.88 (1.76, 2.01) | 1.47 (1.15, 1.87) | 1.88 (1.62, 2.19) | 2.30 (2.13, 2.49) | 1.35 (1.11, 1.64) | 1.34 (1.23, 1.47) |
| Other adult-onset disorder | 1.31 (1.05, 1.65) | 1.02 (0.80, 1.31) | 1.40 (0.75, 2.59) | 1.60 (1.09, 2.36) | 1.44 (1.14, 1.82) | 1.22 (0.74, 2.00) | 0.86 (0.64, 1.16) |
| Childhood-onset disorder | 1.05 (0.54, 2.07) | 1.84 (1.23, 2.74) | 1.95 (0.50, 7.59) | 0.47 (0.07, 3.19) | 1.14 (0.57, 2.26) | 1.43 (0.47, 4.34) | 1.13 (0.66, 1.93) |
| No mental disorder | 1.00 | 1.00 | 1.00 | 1.00 | 1.00 | 1.00 | 1.00 |

*aRR, adjusted relative risk; SGA, small-for-gestational age (birthweight <10th centile for sex and gestation)*

*Models were adjusted for socio-demographic characteristics (parity, maternal age, area based socioeconomic status, area of residence, marital status, and year of birth) and pre-existing conditions (asthma, diabetes, and hypertension)*

Table S5. The associations between maternal mental disorders and perinatal outcomes among Aboriginal women: restricted to one birth per woman (*N =17,177*), Western Australia

|  | aRR (95% CI) | | | | | | |
| --- | --- | --- | --- | --- | --- | --- | --- |
| Women with: | Preterm birth | SGA | Perinatal death | Major birth defect | Low birthweight (<2500g) | Apgar score (<7) at 5 minutes | Fetal distress |
| *Within 5 years before birth* |  |  |  |  |  |  |  |
| Any mental disorder | 1.40 (1.28,1.53) | 1.59 (1.47,1.71) | 1.35 (1.03,1.76) | 1.51 (1.29,1.77) | 1.85 (1.69,2.02) | 1.36 (1.13,1.63) | 1.12 (1.03,1.22) |
| Severe mental disorder | 1.35 (1.04,1.76) | 1.35 (1.05,1.73) | 1.07 (0.45,2.55) | 1.75 (1.15,2.67) | 1.62 (1.24,2.13) | 1.79 (1.09,2.92) | 1.07 (0.82,1.40) |
| Common mental disorder | 1.30 (1.14,1.47) | 1.34 (1.20,1.49) | 1.10 (0.73,1.66) | 1.19 (0.95,1.51) | 1.52 (1.33,1.73) | 1.10 (0.83,1.46) | 1.06 (0.94,1.20) |
| Personality disorder | 1.20 (0.82,1.76) | 1.30 (0.92,1.83) | 0.86 (0.21,3.50) | 1.46 (0.78,2.75) | 1.28 (0.83,1.97) | 1.08 (0.45,2.63) | 1.18 (0.86,1.62) |
| Substance use disorder | 1.53 (1.38,1.70) | 1.83 (1.69,2.00 | 1.44 (1.05,1.97) | 1.86 (1.57,2.21) | 2.21 (2.01,2.44) | 1.51 (1.22,1.87) | 1.24 (1.12,1.37) |
| Other adult-onset disorder | 1.05 (0.86,1.29) | 1.50 (1.29,1.73) | 1.47 (0.88,2.46) | 1.06 (0.75,1.51) | 1.43 (1.19,1.74) | 1.19 (0.81,1.75) | 0.89 (0.74,1.08) |
| Childhood-onset disorder | 1.50 (0.97,2.31) | 1.76 (1.28,2.42) | 3.37 (1.41,8.06) | 1.38 (0.58,3.28) | 1.66 (1.06,2.61) | 1.78 (0.83,3.82) | 0.95 (0.61,1.48) |
| No mental disorder | 1.00 | 1.00 | 1.00 | 1.00 | 1.00 | 1.00 | 1.00 |
| *Within 1 year before birth* |  |  |  |  |  |  |  |
| Any mental disorder | 1.48 (1.32,1.65) | 1.59 (1.44,1.74) | 1.49 (1.06,2.09) | 1.65 (1.36,2.01) | 1.94 (1.74,2.15) | 1.58 (1.26,1.99) | 1.17 (1.05,1.31) |
| Severe mental disorder | 1.39 (0.96,2.02) | 1.42 (1.02,1.98) | 1.75 (0.68,4.47) | 1.42 (0.73,2.78) | 1.78 (1.25,2.53) | 2.80 (1.61,4.85) | 1.21 (0.86,1.71) |
| Common mental disorder | 1.43 (1.20,1.71) | 1.20 (1.01,1.42) | 1.32 (0.76,2.31) | 1.42 (1.04,1.94) | 1.43 (1.19,1.73) | 1.22 (0.82,1.82) | 1.02 (0.85,1.22) |
| Personality disorder | 1.54 (0.94,2.53) | 1.35 (0.80,2.27) | 1.05 (0.15,7.50) | 1.96 (0.86,4.49) | 1.41 (0.77,2.58) | 1.06 (0.27,4.23) | 1.35 (0.88,2.09) |
| Substance use disorder | 1.55 (1.36,1.78) | 1.90 (1.71,2.11) | 1.41 (0.92,2.15) | 2.01 (1.61,2.50) | 2.34 (2.08,2.64) | 1.63 (1.23,2.15) | 1.28 (1.12,1.46) |
| Other adult-onset disorder | 1.19 (0.81,1.75) | 0.89 (0.59,1.35) | 1.35 (0.43,4.30) | 1.64 (0.90,2.98) | 1.22 (0.80,1.86) | 1.26 (0.57,2.81) | 0.75 (0.47,1.19) |
| Childhood-onset disorder | 1.43 (0.70,2.93) | 2.41 (1.58,3.67) | 3.63 (0.93,14.12) | NA | 1.70 (0.83,3.48) | 1.63 (0.46,5.75) | 1.17 (0.63,2.19) |
| No mental disorder | 1.00 | 1.00 | 1.00 | 1.00 | 1.00 | 1.00 | 1.00 |

*aRR, adjusted relative risk; SGA, small-for-gestational age (birthweight <10^th^ centile for sex and gestation)*

*Models were adjusted for socio-demographic characteristics (parity, maternal age, area based socioeconomic status, area of residence, marital status, and year of birth) and pre-existing conditions (asthma, diabetes, and hypertension)*

Table S6. The associations between maternal mental disorders and preterm and SGA categories among births to Aboriginal women: restricted to one birth per woman (N =17,177), Western Australia

|  | aRR (95% CI) | | | |
| --- | --- | --- | --- | --- |
| Women with: | Term-AGA | Preterm-SGA | Preterm-AGA | Term-SGA |
| *Within 5 years before birth* |  |  |  |  |
| Any mental disorder | 1.00 | 2.79 (2.18,3.57) | 1.55 (1.38,1.75) | 1.83 (1.65,2.03) |
| Severe mental disorder | 1.00 | 2.47 (1.22,4.97) | 1.44 (1.00,2.07) | 1.42 (1.01,2.00) |
| Common mental disorder | 1.00 | 2.19 (1.55,3.11) | 1.36 (1.15,1.61) | 1.42 (1.23,1.66) |
| Personality disorder | 1.00 | 1.45 (0.45,4.71) | 1.32 (0.79,2.21) | 1.44 (0.90,2.29) |
| Substance use disorder | 1.00 | 3.54 (2.67,4.68) | 1.83 (1.58,2.11) | 2.30 (2.03,2.61) |
| Other adult-onset disorder | 1.00 | 2.28 (1.41,3.68) | 1.02 (0.78,1.33) | 1.60 (1.30,1.96) |
| Childhood-onset disorder | 1.00 | 4.86 (1.88,12.58) | 1.56 (0.82,2.97) | 2.03 (1.20,3.41) |
| No mental disorder | 1.00 | 1.00 | 1.00 | 1.00 |
| *Within 1 year before birth* |  |  |  |  |
| Any mental disorder | 1.00 | 3.60 (2.71,4.78) | 1.60 (1.36,1.88) | 1.78 (1.54,2.05) |
| Severe mental disorder | 1.00 | 2.33 (0.83,6.55) | 1.62 (0.96,2.73) | 1.62 (1.01,2.62) |
| Common mental disorder | 1.00 | 2.44 (1.52,3.92) | 1.49 (1.17,1.89) | 1.22 (0.96,1.54) |
| Personality disorder | 1.00 | 3.61 (1.07,12.17) | 1.58 (0.73,3.39) | 1.31 (0.59,2.87) |
| Substance use disorder | 1.00 | 4.73 (3.42,6.55) | 1.79 (1.47,2.19) | 2.35 (1.99,2.79) |
| Other adult-onset disorder | 1.00 | 2.72 (1.16,6.34) | 0.95 (0.55,1.64) | 0.65 (0.36,1.17) |
| Childhood-onset disorder | 1.00 | 6.69 (1.42,31.50) | 1.87 (0.57,6.06) | 3.84 (1.61,9.13) |
| No mental disorder | 1.00 | 1.00 | 1.00 | 1.00 |

*aRR, adjusted relative risk; AGA, appropriate-for-gestational age; SGA, small-for-gestational age (birthweight <10th centile for sex and gestation)*

*Models were adjusted for socio-demographic characteristics (parity, maternal age, area based socioeconomic status, area of residence, marital status, and year of birth) and pre-existing conditions (asthma, diabetes, and hypertension)*

Table S7. The associations between maternal mental disorders and any adverse perinatal outcome among births to Aboriginal women, Western Australia^a^

| Women with: | Sample 1  (Main analysis)  *N=38,592* | Sample 2 (Imputed covariate with missing data)  *N=40,536* | Sample 3 (Imputed all variables with missing data)  *N=44,167* | Sample 4 (Restricted to one birth per woman)  N*=17,177* |
| --- | --- | --- | --- | --- |
|  | aRR (95% CI) | aRR (95% CI) | aRR (95% CI) | aRR (95% CI) |
| *Within 5 years before birth* |  |  |  |  |
| Any mental disorder | 1.30 (1.27,1.34) | 1.30 (1.26, 1.34) | 1.30 (1.26, 1.34) | 1.27 (1.22, 1.32) |
| Severe mental disorder | 1.23 (1.12,1.36) | 1.25 (1.14, 1.37) | 1.26 (1.15, 1.38) | 1.25 (1.11, 1.41) |
| Common mental disorder | 1.22 (1.17,1.27) | 1.21 (1.16, 1.26) | 1.20 (1.16, 1.26) | 1.16 (1.09, 1.23) |
| Personality disorder | 1.22 (1.06,1.40) | 1.23 (1.08, 1.42) | 1.22 (1.06, 1.40) | 1.21 (1.02, 1.43) |
| Substance use disorder | 1.41 (1.37,1.46) | 1.41 (1.37, 1.46) | 1.41 (1.37, 1.46) | 1.40 (1.34, 1.47) |
| Other adult-onset disorder | 1.24 (1.17,1.31) | 1.23 (1.16, 1.30) | 1.22 (1.16, 1.30) | 1.11 (1.02, 1.21) |
| Childhood-onset disorder | 1.21 (1.04,1.40) | 1.19 (1.02, 1.38) | 1.19 (1.03, 1.39) | 1.30 (1.08, 1.56) |
| No mental disorder | 1.00 | 1.00 | 1.00 | 1.00 |
| *Within 1 year before birth* |  |  |  |  |
| Any mental disorder | 1.36 (1.31,1.41) | 1.36 (1.31, 1.41) | 1.36 (1.31, 1.40) | 1.30 (1.23, 1.36) |
| Severe mental disorder | 1.36 (1.20,1.53) | 1.38 (1.22, 1.55) | 1.39 (1.24, 1.56) | 1.33 (1.14, 1.56) |
| Common mental disorder | 1.23 (1.16,1.30) | 1.23 (1.16, 1.30) | 1.22 (1.16, 1.29) | 1.16 (1.06, 1.26) |
| Personality disorder | 1.35 (1.11,1.63) | 1.38 (1.15, 1.66) | 1.36 (1.13, 1.63) | 1.28 (1.01, 1.62) |
| Substance use disorder | 1.49 (1.43,1.55) | 1.49 (1.44, 1.55) | 1.49 (1.43, 1.54) | 1.42 (1.34, 1.50) |
| Other adult-onset disorder | 1.07 (0.94,1.22) | 1.05 (0.93, 1.20) | 1.06 (0.93, 1.20) | 0.87 (0.69, 1.09) |
| Childhood-onset disorder | 1.19 (0.92,1.55) | 1.18 (0.90, 1.54) | 1.21 (0.94, 1.56) | 1.35 (1.03, 1.77) |
| No mental disorder | 1.00 | 1.00 | 1.00 | 1.00 |

*aRR, adjusted relative risk; CI, confidence interval*

*^a^Any adverse perinatal outcome was defined as the occurrence of any of the following: preterm birth, small-for-gestational age (birthweight <10^th^ centile for sex and gestation), perinatal death, congenital anomaly, low birthweight, low Apgar score at 5 minutes or fetal distress.*

*Models were adjusted for socio-demographic characteristics (parity, maternal age, area based socioeconomic status, area of residence, marital status, and year of birth) and pre-existing conditions (asthma, diabetes, and hypertension).*
